# Statistical Effective Diffusivity Estimation in Porous Media Using an Integrated On-site Imaging Workflow for Synchrotron Users

**DOI:** 10.1007/s11242-023-01993-7

**Published:** 2023-07-26

**Authors:** James Le Houx, Siul Ruiz, Daniel McKay Fletcher, Sharif Ahmed, Tiina Roose

**Affiliations:** 1 Department, Diamond Light Source, Harwell Science and Innovation Campus, Fermi Ave, Didcot, Oxfordshire OX11 0DE UK; 2 Faculty of Engineering and Physical Sciences, University of Southampton, University Road, Southampton, Hampshire SO17 1BJ UK; 3 Rural Economy, Environment and Society, Scotland’s Rural College, West Mains Road, Edinburgh, EH9 3JG UK

**Keywords:** Porous media, Image-based modelling, Homogenisation, Numerical methods

## Abstract

Transport in porous media plays an essential role for many physical, engineering, biological and environmental processes. Novel synchrotron imaging techniques and image-based models have enabled more robust quantification of geometric structures that influence transport through the pore space. However, image-based modelling is computationally expensive, and end users often require, while conducting imaging campaign, fast and agile bulk-scale effective parameter estimates that account for the pore-scale details. In this manuscript we enhance a pre-existing image-based model solver known as OpenImpala to estimate bulk-scale effective transport parameters. In particular, the boundary conditions and equations in OpenImpala were modified in order to estimate the effective diffusivity in an imaged system/geometry via a formal multi-scale homogenisation expansion. Estimates of effective pore space diffusivity were generated for a range of elementary volume sizes to estimate when the effective diffusivity values begin to converge to a single value. Results from OpenImpala were validated against a commercial finite element method package COMSOL Multiphysics (abbreviated as COMSOL). Results showed that the effective diffusivity values determined with OpenImpala were similar to those estimated by COMSOL. Tests on larger domains comparing a full image-based model to a homogenised (geometrically uniform) domain that used the effective diffusivity parameters showed differences below 2 % error, thus verifying the accuracy of the effective diffusivity estimates. Finally, we compared OpenImpala’s parallel computing speeds to COMSOL. OpenImpala consistently ran simulations within fractions of minutes, which was two orders of magnitude faster than COMSOL providing identical supercomputing specifications. In conclusion, we demonstrated OpenImpala’s utility as part of an on-site tomography processing pipeline allowing for fast and agile assessment of porous media processes and to guide imaging campaigns while they are happening at synchrotron beamlines.

**Supplementary Information:**

The online version contains supplementary material available at 10.1007/s11242-023-01993-7.

## Introduction

Advancements in precision engineering and imaging technologies have enabled studies of porous media in unprecedented detail than ever before. In particular, micro-X-ray computed tomography (XRCT) has shown great promise in enhancing understanding of how pore-scale structures influence mass transport (Keyes et al. 2013; Koebernick et al. 2019). XRCT now routinely provides 3D geometric features at the micron scales. By analysing these features, researchers have developed simple morphological metrics that provide qualitative insights into how pore-scale features influence bulk-scale processes (Vogel 2002) such as water and nutrient transport in soils (Vereecken et al. 2016). Understanding these transport processes can aid in improving agricultural practices through enhanced fertiliser use efficiency (Duncan et al. 2018) and enhancing agricultural yields, thus mitigating many issues associated with food security and environmental pollution. However, making scientific inferences based on imaged structural data alone is difficult without inclusion of the physical process governing the phenomena (Vereecken et al. 2016).

XRCT data sets are often large, with single tomographies producing images containing $10^{10}$ voxels, resulting in 40GB of data for a single scan. Experiments often require tens to hundreds of scans, and data analyses for these scans are expensive in terms of time and computing resources. It is difficult to assess what resolution is representative of a given domain or scientific problem prior to image acquisition. Furthermore, structural images alone seldom provide sufficient information about physical processes such as chemical reactions or biological activity (Keyes et al. 2022). As such, XRCT images often have to be coupled with auxiliary measurements or supplemented with specific theoretical models and computations.

The technique of image-based modelling (i.e. using the 3D XRCT image as a physical modelling domain) provides a beneficial avenue to supplement XRCT imaging (Ruiz et al. 2021). Image-based models create a digital replica (i.e. digital twin) of the physical setup that often is impossible to investigate experimentally. Thus, these simulations act as *in silico* experiments. They enable rigorous quantification of processes occurring in the physical systems, which enables the extraction of functional results from structural imaging data sets. Furthermore, image-based modelling enables researchers to generate inferences on quantities or processes otherwise unmeasurable by conventional experimental techniques (Ruiz et al. 2020).

In order to generate image-based models on large XRCT data sets, computational and analytic methods are often used to reduce the computational infrastructure load. These methods involve downsampling data at the cost of image resolution. Another approach is to select a representative elementary volume (REV), which can be potentially subjective. Other methods discretise the images via a pore network modelling approach (Yang et al. 2019; Callow et al. 2020). Thus, most studies do not require all of the pore-scale details and would suffice with a practical description of the modelled domain.

Alternatively, a multi-scale asymptotic homogenisation approach may be used to extract effective parameters to avoid running several computationally expensive image-based models. This method has been used in soil physics studies to formally derive Darcy’s law (Hornung 1996) and upscale partially water saturated flow on a representative elementary volume (Cooper et al. 2017). The method ultimately extracts effective parameters that account for necessary pore-scale heterogeneities on a large-scale domain. Thus, the effective parameters are often representative of what studies have measured at bulk scales. As such, there is value in obtaining effective parameters that describe an imaged system at larger scales while simultaneously obtaining images at a beamline as this will allow for live functional characterisation of the imaged samples and possible changes in sampling and imaging.

The dual imaging and diffraction (DIAD) beamline at the diamond light source (Reinhard et al. 2021) is developing an open-source simulation package named OpenImpala that endeavours to provide users with bulk-scale effective transport parameter estimates on-site. OpenImpala is a highly parallelisable finite difference package (FDM) for solving equilibrium partial differential equations (PDEs) directly on image domains which can leverage high-performance computing infrastructures for large data sets associated with XRCT data. In this study, we adapt OpenImpala to efficiently extract effective transport properties from XRCT images, with the goal of being able to perform simulations at rates that could be conducted while a user is at the beamline. As such, the objective of this paper is to:

- Update OpenImpala such that it generates estimates for effective diffusive transport properties used in homogenised approaches
- Generate comparisons between OpenImpala’s estimates and a commercial benchmark finite element model (FEM) for varying elementry volume (EV) size
- Use the time-dependent FEM solver to estimate the error between the effective parameters in the homogenised model and the full image-based model
- Determine time efficiency when using OpenImpala as opposed to the commercial FEM package

We will start by providing a brief overview of the theoretical framework used in the study. We will then describe the imaged domain used for generating effective parameter estimates for varying EVs. Subsequently, we generate a periodically repeating imaged domain to test the efficacy of the effective parameter. Lastly, we will compare the computational time between OpenImpala and the commercial FEM package and discuss similarities and differences in the context of different scientific disciplines.

## Theoretical Consideration

### Mathematical Homogenisation Method

#### General Overview

**Fig. 1:**
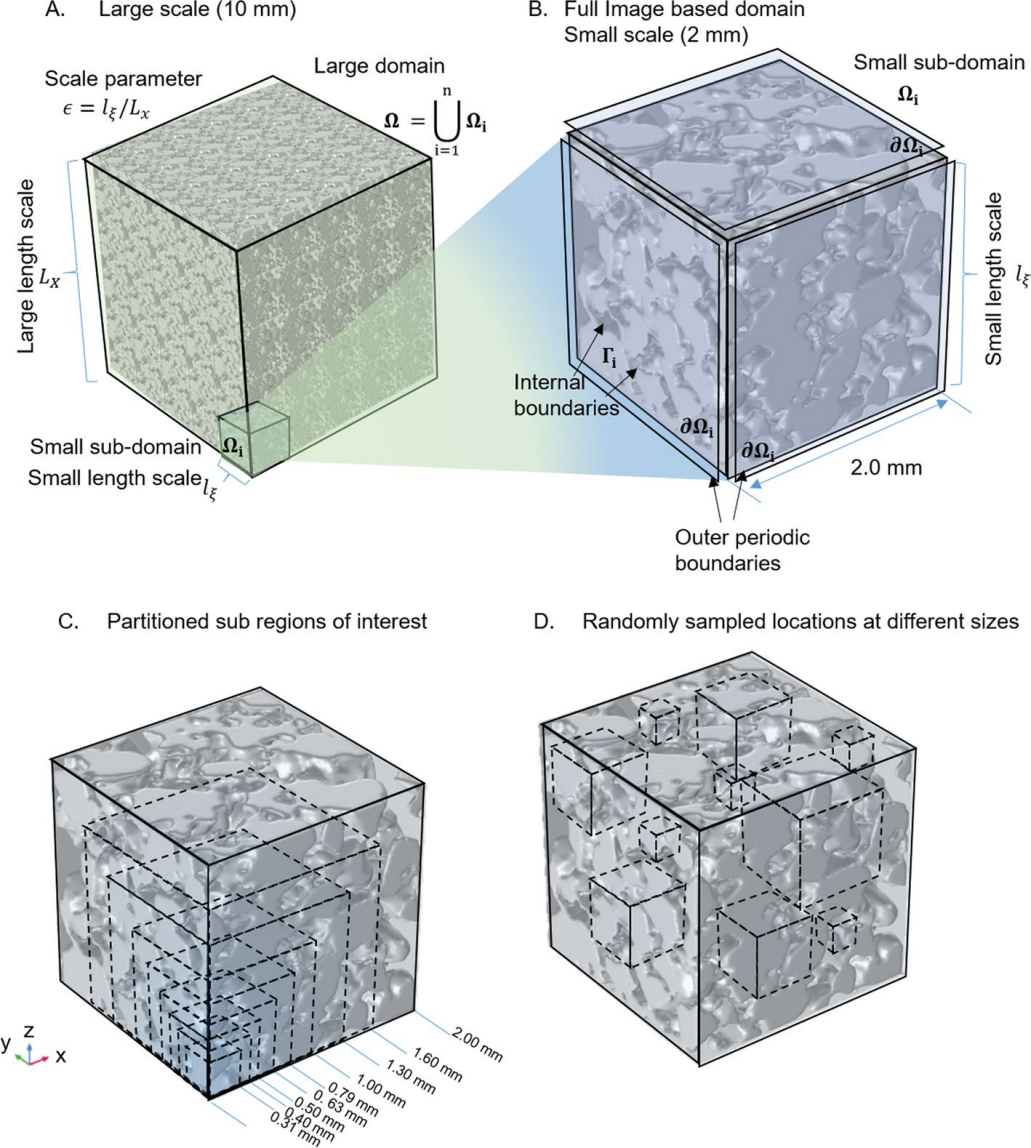
Generating effective parameter estimate based on XCT images. **A** Representation of a large-scale pore space $\Omega$ comprised of periodic subdomains $\Omega _i$. The length scale of the large domain is represented by $L_X$ while the length scale of the subdomain is represented by $l_\xi$. **B** The full image-based geometry representing the pore space within a porous media (soil pore space in the given example). **C** Determining the representative elementary volume sufficient for obtaining reasonable effective parameter (i.e. diffusivity) estimates. **D** Generating a region of interest for a given size at a random location in the modelled domain

Quantifying mass transport of solutes in domains with complex geometries can be computationally expensive or even unfeasible. In many fields associated with porous media (e.g. soil science and electrochemistry), it is often more productive and informative to obtain effective transport parameters for a bulk domain rather than include all of the nuanced geometric complexities (Daly et al. 2016; Hack et al. 2020). This process only works if we look at the problem solution on the scale of a collection of many different particles or pores. Such formal effective parameter estimation for different physical phenomena in porous media is well established (Hornung 1996). This current paper focuses on modelling the diffusive transport of solutes in the porous media domain that is obtained from imaging. As stated, a critical assumption that will be followed in this work is that there exists a separation of scales associated with the large-scale processes, i.e. it is associated with the large length scale $L_X$ and the small scale associated with the small length scale $l_{\xi }$ (Fig. 1 A). The small scale presents all of the imaged detail in the pore space and is assumed mathematically to be repeating, and hence (semi-)periodic, in the different directions (Fig. 1 A–B). The large-scale domain modelling problem can then take the estimated effective (or homogenised) diffusivity and generate estimates sufficient for describing the large transport scale behaviour without explicitly considering all pore-scale geometric detail.

#### Non-dimensionalising the Diffusion Equation

Here, we will only present the essential description needed to estimate effective parameters (i.e. diffusivity). For an in-depth derivation, see SI. Considering the diffusion equation:

1 $$\begin{aligned} \frac{\partial {\tilde{c}}}{\partial {\tilde{t}}} = {\tilde{\nabla }} \cdot ({\tilde{D}} {\tilde{\nabla }} {\tilde{c}}), \; {\tilde{\textbf{x}}} \in \Omega , \end{aligned}$$

where ${\tilde{c}}$ [mol m$^{-3}$] is a concentration of a transporting solute, ${\tilde{t}}$ [s] is time, ${\tilde{D}}$ [m$^2$ s$^{-1}$] is the diffusivity of the solute in liquid, ${\tilde{\textbf{x}}}$ is the spatial coordinate system associated with regions in the domain volume $\Omega$ (Fig. 1 A), and ${\tilde{\nabla }}$ is the spatial differentiation operator. We non-dimensionalise the equation with the following scalings

2 $$\begin{aligned} \begin{array}{ll} {\tilde{\nabla }}(\cdot ) = \frac{1}{L_X} \nabla (\cdot ), &{} {\tilde{t}} = \tau t \\ {\tilde{c}} = {\bar{c}} c, &{} {\tilde{D}} = \frac{L_{x}^2}{\tau } D \end{array}, \end{aligned}$$

where $L_X$ [m] is the large length scale, $\tau$ [s] is the time scale, and ${\bar{c}}$ is the average initial concentration of solute. The non-dimensional diffusion equation is now given by

3 $$\begin{aligned} \frac{\partial c}{\partial t} = \nabla \cdot (D \nabla c), \; {\textbf{x}} \in \Omega . \end{aligned}$$

We assume that the domain $\Omega = \bigcup _i^n \Omega _i$, where each $\Omega _i$ is a periodically repeating sub-domain (or cell) (Fig. 1 A–B). As such, the boundary condition for periodicity is given as:

4 $$\begin{aligned} c -\; periodic, \; {\textbf{x}} \in \partial \Omega _i, \end{aligned}$$

where $\partial \Omega _i$ is the outer boundary of a cell (Fig. 1 B). We assume that solutes cannot move across internal boundaries between pore-space and solids:

5 $$\begin{aligned} {\hat{\textbf{n}}} \cdot (D \nabla c)=0, \; {\textbf{x}} \in \Gamma _i, \end{aligned}$$

where $\Gamma _i$ represents the internal boundaries between solids and pore-space (Fig. 1B).

#### Multi-scale Expansion

The dimensional differential operator can be expanded into a large scale estimate and a small-scale corrector of the spatial derivative

6 $$\begin{aligned} {\tilde{\nabla }}(\cdot ) = {\tilde{\nabla }}_{X}(\cdot ) + {\tilde{\nabla }}_{\xi }(\cdot ), \end{aligned}$$

where ${\textbf{X}}$ and $\varvec{\xi }$ are the large and small scale spatial variable, respectively. The dimensional scaling of the differential operators is given by:

7 $$\begin{aligned} \begin{array}{ll} {\tilde{\nabla }}_{X}(\cdot ) = \frac{1}{L_X} \nabla _{X}(\cdot ),&{\tilde{\nabla }}_{\xi }(\cdot ) = \frac{1}{l_{\xi }} \nabla _{\xi }(\cdot ) \end{array}, \end{aligned}$$

Considering the relationship between the large scale and small scale as $L_X>> l_{\xi }$, we say that the ratio between the scales is given by $\frac{l_{\xi }}{L_x} = \epsilon$ where $\epsilon$ is a small number. Our dimensionless differential operator, assuming these scales are independent, can be expressed using chain rule as

8 $$\begin{aligned} \nabla (\cdot ) = \nabla _X(\cdot )+\epsilon ^{-1}\nabla _{\xi }(\cdot ). \end{aligned}$$

We then assume that the concentration can be expanded in power of $\epsilon$ and assuming that concentration *c* is dependent $\xi$ and *X* independently we get

9 $$\begin{aligned} c = \epsilon ^{0}c_{(0)}(X,\xi ,t)+\epsilon ^{1}c_{(1)}(X,\xi ,t)+\epsilon ^{2}c_{(2)} (X,\xi ,t)+{\mathcal {O}}(\epsilon ^3) \end{aligned}$$

Substituting in Eqs. 8 and 9 in Eq. 3 (see SI for full workings), we obtain the following equation on the cell:

10 $$\begin{aligned} \epsilon ^{0} \frac{{\partial c_{{(0)}} }}{{\partial t}} = \;& \epsilon ^{{ - 2}} \nabla _{\xi } \cdot \left( {D\nabla _{\xi } c_{{(0)}} } \right) + \epsilon ^{{ - 1}} (\nabla _{\xi } \cdot (D(\nabla _{\xi } c_{{(1)}} + \nabla _{X} c_{{(0)}} )) \\ & + \nabla _{X} \cdot (D\nabla _{\xi } c_{{(0)}} )) + \epsilon ^{0} (\nabla _{\xi } \cdot (D(\nabla _{\xi } c_{{(2)}} + \nabla _{X} c_{{(1)}} )) \\ & + \nabla _{X} \cdot (D(\nabla _{\xi } c_{{(1)}} + \nabla _{X} c_{{(0)}} )) + {\mathcal{O}}( \epsilon ^{1} ),\xi \in \Omega _{i} , \\ \end{aligned}$$

with the associated no flux conditions:

11 $$\begin{aligned} \begin{array}{ll} \epsilon ^{-2} {\hat{\textbf{n}}} \cdot (D \nabla _{\xi } c_{(0)}) + \epsilon ^{-1}( {\hat{\textbf{n}}} \cdot (D (\nabla _{\xi } c_{(1)}+\nabla _{X} c_{(0)}))) \\ + \epsilon ^{0}( {\hat{\textbf{n}}} \cdot (D (\nabla _{\xi } c_{(2)}+\nabla _{X} c_{(1)}))) +{\mathcal {O}}(\epsilon ^1) = 0, &{} \varvec{\xi }\in \Gamma _i, \end{array} \end{aligned}$$

and periodic boundary conditions

12 $$\begin{aligned} c_{(0)}, c_{(1)}, c_{(2)},... -\; periodic, \; \varvec{\xi }\in \partial \Omega _i. \end{aligned}$$

**Fig. 2:**
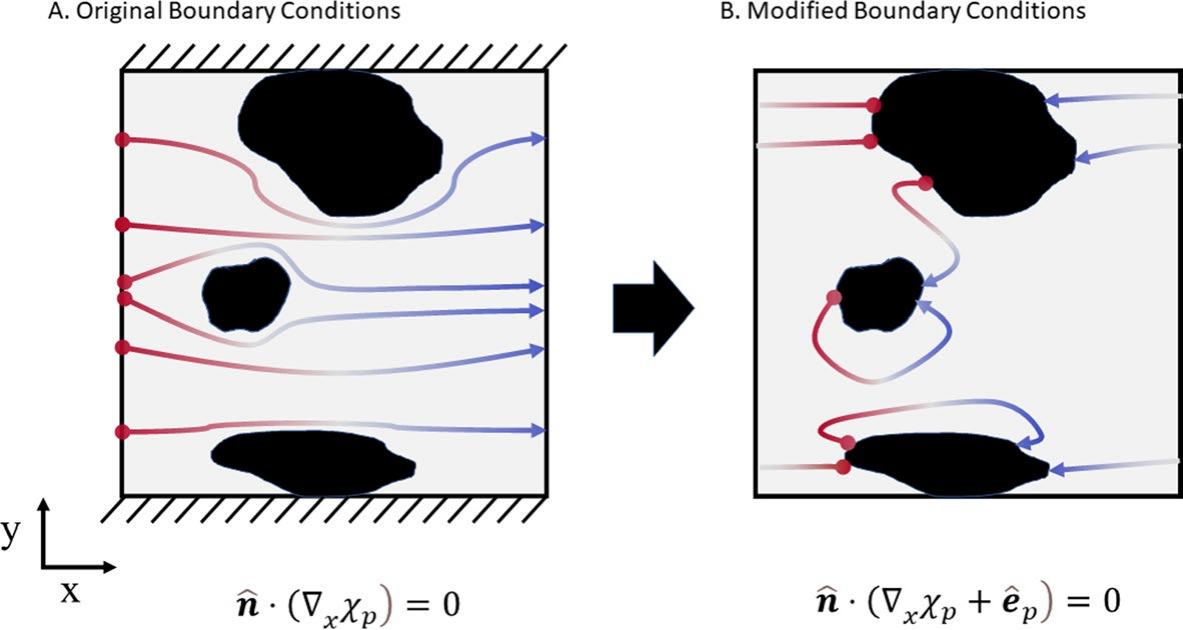
Visual representation of the modification of OpenImpala’s boundary conditions for the cell problem. **A** Dirichlet input and output conditions on opposing faces, with zero flux implemented on the remaining faces, as detailed in Le Houx and Kramer (2021). **B** Modified boundary conditions where input and output Neumann conditions are implemented on opposite sides of the non-porous particles, the remaining walls use a periodic boundary condition

We note that given the periodicity of the cell, it is sufficient to consider $D = D(\varvec{\xi })$. We split the equations in accordance with the different orders ${\mathcal {O}}(\epsilon ^{n})$ and extract specific information from each order of $\epsilon$ (see SI for more details). To summarise, the order ${\mathcal {O}}(\epsilon ^{-2})$ equations inform us that $c_{(0)}(t, \varvec{\xi },{\textbf{X}}) = c_{(0)}(t,{\textbf{X}})$, thus not varying on the small scale. The order ${\mathcal {O}}(\epsilon ^{-1})$ equations are used to generate the system of equations whose solutions will act as small-scale corrections to the large-scale solution

13 $$\begin{aligned} \left\{ \begin{array}{ll} \nabla _{\xi } \cdot (D \nabla _{\xi } \chi _k) = - \nabla _{\xi } \cdot (D {\hat{\textbf{e}}}_i), &{} \quad \varvec{\xi }\in \Omega _i \\ {\hat{\textbf{n}}} \cdot (D (\nabla _{\xi } \chi _k+{\hat{\textbf{e}}}_i))=0, &{} \quad \varvec{\xi }\in \Gamma _i \\ \chi _k - \; periodic, &{} \quad \varvec{\xi }\in \partial \Omega _i \end{array} \right. , \end{aligned}$$

where $\chi _k$ is the small-scale corrector in the $k^{th}$ direction (see Fig. 2 for details). Finally, we can determine our homogenised equation on the order ${\mathcal {O}}(\epsilon ^{0})$ equations:

14 $$\begin{aligned} \frac{\partial c_{(0)}}{\partial t} = \nabla _{X} \cdot ( {\mathfrak {D}}_{eff} \nabla _{X} c_{(0)}), \; \; {\textbf{X}} \in \Omega , \end{aligned}$$

where ${\mathfrak {D}}_{eff}$ is the 3-by-3 effective diffusivity tensor (where diagonal entries define the effective diffusion in each direction) and depends on the solutions of the cell problems (Eq. 13):

15 $$\begin{aligned} {\mathfrak {D}}_{eff} = \frac{1}{\Vert \Omega _i\Vert } \int _{\Omega _i}{ (D (\nabla _{\xi }\chi _k \otimes {\hat{\textbf{e}}}_k+{\textbf{I}}) ) d\varvec{\xi }}, \end{aligned}$$

where ${\textbf{I}}$ is the 3-by-3 identity tensor and $\nabla _{\xi }\chi _k \otimes {\hat{\textbf{e}}}_k = \sum _{i=1}^n{\nabla _{\xi }\chi _k {\hat{\textbf{e}}}_i^{T}}$. We emphasise that a representative image-based volume is needed for only the cell problem (Eq. 13), which is a steady state equation. The solution from that problem can then be applied and upscaled for much larger homogenised domains, subsequently alleviating the need to simulate large geometrically complex problems and ultimately reducing the net computational time by orders of magnitude (Duncan et al. 2018).

## Methods

### Homogenisation of Elementary Volumes (EVs) of an Image-Based Model

In this study, we take a detailed pore space segmentation from XRCT scans obtained from the TOMCAT beamline at the Paul Scherrer Institute, Villigen Switzerland (Fig. 1B) Daly et al. (2016) and generate model domains based on these images. The full domain represents a 2 mm soil pore space (Daly et al. 2016). We partition the pore space into 8 concentric EVs and run simulations on each of the domains ((Fig. 1C). For each domain size, we calculate the effective diffusivity using Eq. 15.

The effective parameter is calculated numerically using the finite difference method (FDM) in OpenImpala and the finite element method (FEM) in COMSOL Multiphysics (abbreviated as COMSOL) Multiphysics (1998). For FEM simulations, image-based meshes were generated using a software called ScanIP (Johnson and Officer 2005). A relatively fine mesh specification was used since the FEM solution was to act as benchmark. The full domain mesh comprised of 899,248 tetrahedral elements and meshes of subvolumes were meshed to obtain a similar volume-to-mesh-element ratio and mesh quality. This mesh quality was chosen to ensure the numerical solution had little error introduced by meshing. Components of the effective diffusivity tensor were reported for the diagonal values (i.e. *x*, *y*, and *z* directions) and the mean values of all the tensor components. Values obtained for FDM and FEM were compared for the different EVs. Convergence behaviour was also monitored for increasing EVs.

Furthermore, using OpenImpala, we generate a region of interest of a fixed size at ten random locations within the small-scale subdomain. For different fixed sizes, we estimate ensemble means of the effective diffusivity and monitor the variability associated with the different-sized regions of interest.

### OpenImpala–Finite Difference

OpenImpala was chosen for the FDM simulations as it is an open-source image-based modelling tool capable of leveraging advantages from HPC infrastructure, such as massive parallelisation (Le Houx et al. 2020; Fraser et al. 2022). OpenImpala uses a labelled XRCT-generated voxel data set to discretise the problem, meaning no additional meshing is required. In order to perform equivalent effective parameter estimation using OpenImpala, it was necessary to modify the computer code to implement the same simulation conditions as is typically performed on representative soil volumes. A 2D visual representation of this modification can be seen in Fig. 2.

Originally, OpenImpala employed a steady-state through-flow type simulation, as discussed in Le Houx and Kramer (2021). For this study, the Dirichlet boundary conditions were imposed on the two opposing inlet and outlet faces, and a no-flux wall condition was imposed on the remaining faces, as seen in Fig. 2A As discussed in Nguyen et al. (2020), flow-through type simulations discount ’dead-end pores’ from the effective parameter estimation, where ’dead-end pores’ are those that do not fully connect across the thickness of the image data set. In the case of soil, porous battery electrodes, supercapacitors and fuel cells, this is not accurate as these samples do not require transport from one side of the structure to the other. Instead, the combination of transport throughout the interconnected volume between different regions contributes to the effective parameter estimation. Thus, OpenImpala’s Fortran kernel was modified to represent the equations derived in Sect. 2.1. In practice, this meant implementing periodic boundary conditions on each domain faces and modifying the inlet and outlet boundaries to opposing sides of non-conducting media, as seen in Fig. 2B The routines developed during this paper have been released within v1.1.0 of OpenImpala on the publicly accessible Github repository, https://github.com/kramergroup/openImpala/.

### Data Processing Pipeline Architecture

**Fig. 3:**
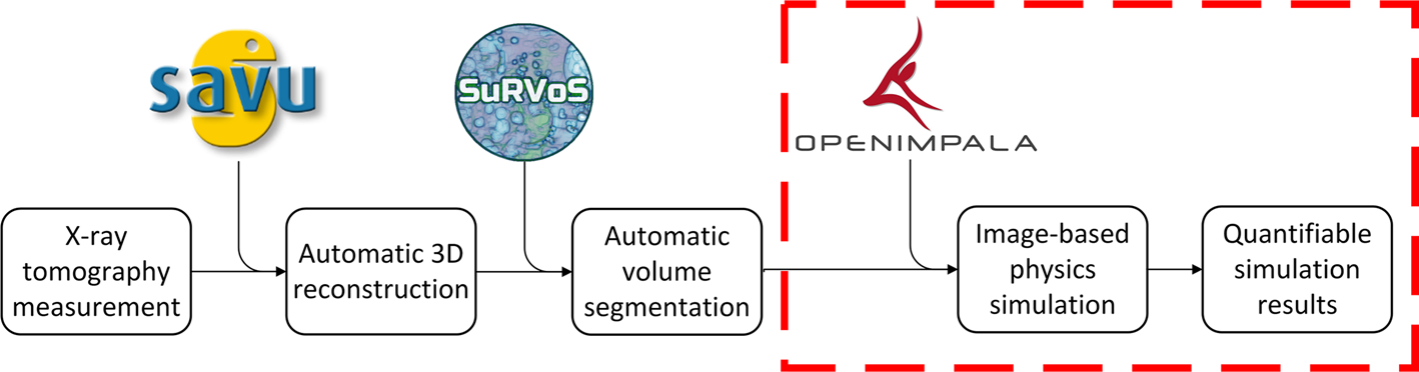
Overview of the semi-automated reconstruction, segmentation and simulation pipeline

The Dual Imaging And Diffraction beamline at the Diamond Light Source (Reinhard et al. 2021) has developed an on-site deployable semi-automated data processing pipeline. As part of this pipeline, X-ray tomography data sets are reconstructed into 3D volumes using a filtered-back projection algorithm through the Python-based package, Savu Wadeson et al. (2016), segmented into constituent phases using a supervised convolutional neural network using SuRVoS (Pennington et al. 2022), and finally, the classified, real 3D domain is used as the basis for an image-based model. These results can then inform experimental procedure while beamline experiments are still running. In order to achieve this, a voxel-based solver is desirable to remove the time and resource-costly meshing step. The methods developed this paper will be used as part of this pipeline and are highlighted in Fig. 3.

### Model Validation of Homogenised Solution Considering Periodically Increasing Domain Length

**Fig. 4:**
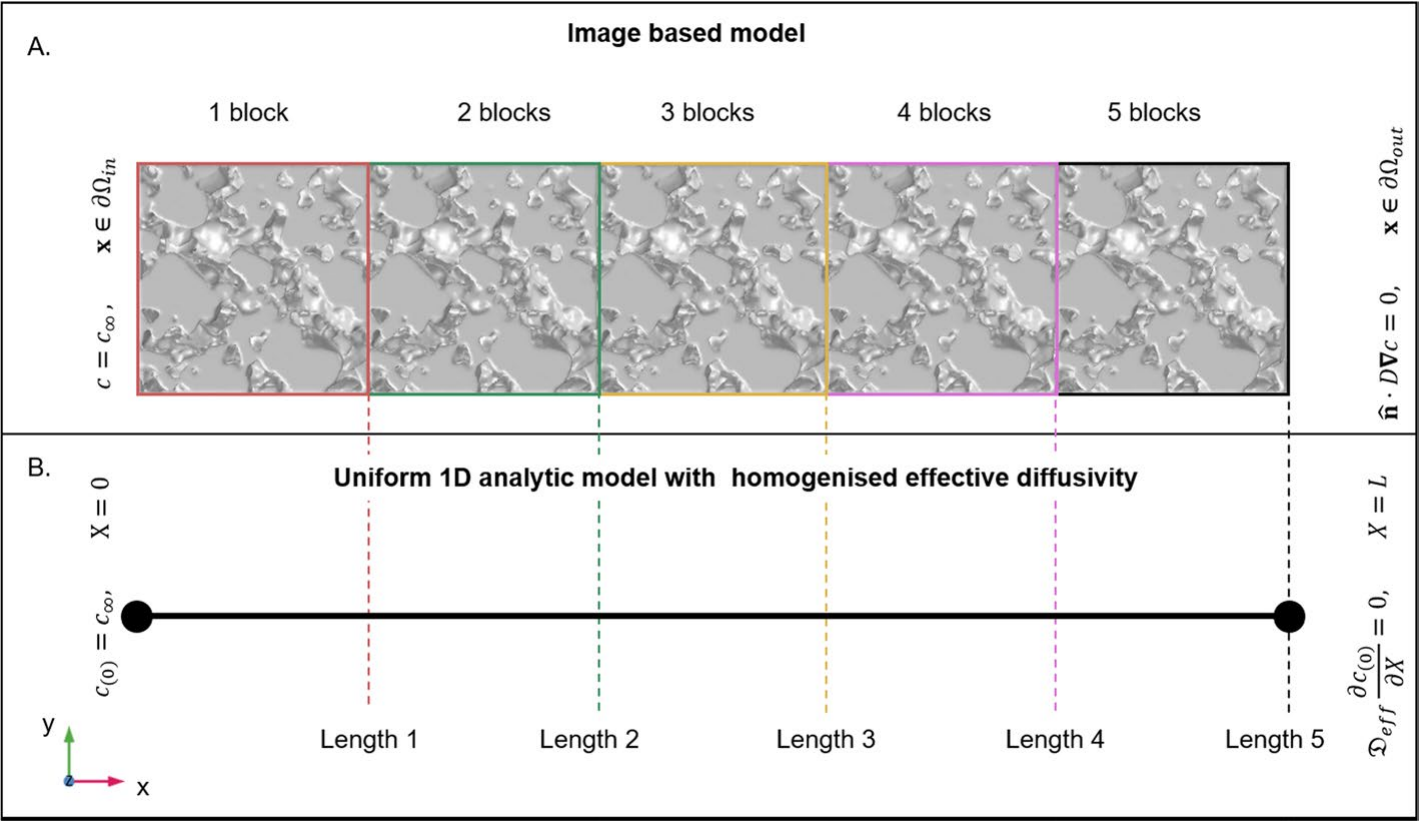
Simulations conducted on **A** Image-based domains and **B** uniform 1D domain for increasing domain length. Simulations were conducted for both on a length associated with a domain length of 1 (red), 2 (green), 3 (yellow), 4 (pink) and 5 (black). Simulations consider a Dirichlet boundary at the far left surface and no flux at the far right end

To validate the homogenisation procedure, we performed a comparison between the image-based simulation and a uniform 1D domain with effective diffusivity parameters using an analytic solution to capture time dependence. The full image-based domain was duplicated along the *x*-direction five times ( Fig. 4A). The simulations were conducted with a fixed concentration on the left hand side and no flux condition on the right hand side. A 1D model with the same lengths was created with the same boundary conditions (see SI section 2 for details). The homogenised effective diffusivity was used in the uniform geometry. For the different lengths, the average concentration was measured and compared between the image-based and uniform 1D homogenised models over time. The maximum differences between the two simulation approaches were used to estimate the error between the two models:

16 $$\begin{aligned} \varepsilon _{\%,i} = \frac{ \Vert \langle c \rangle _i - \langle c_{(0)} \rangle _i \Vert _{\infty }}{\langle c \rangle _i} 100\%, \end{aligned}$$

where $\langle c \rangle _i$ and $\langle c_{(0)} \rangle _i$ are the average concentration of the boundary at length *i*.

## Results

### Homogenisation Comparison Between COMSOL FEM and OpenImpala FDM

**Fig. 5:**
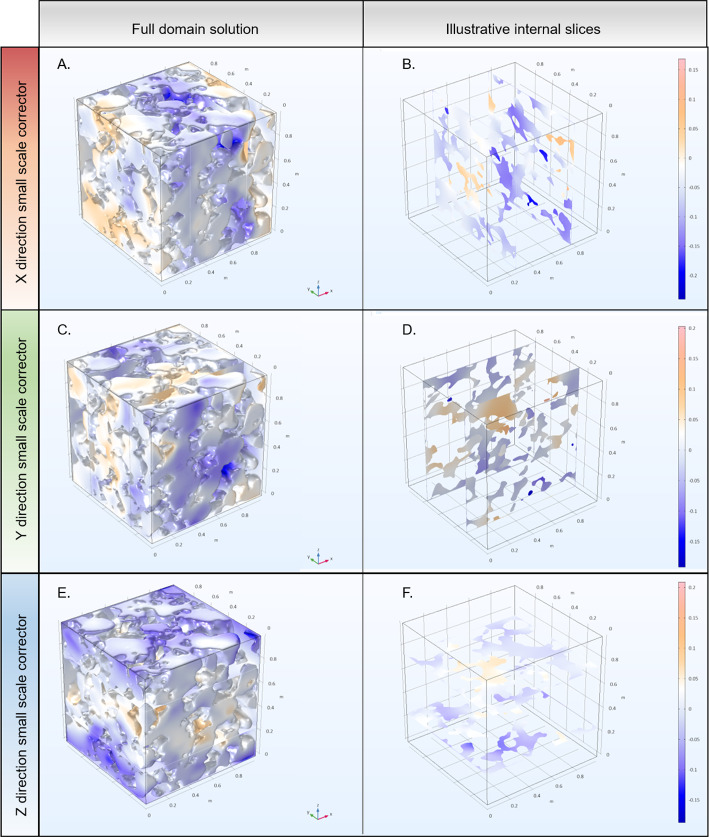
Illustrative results of the cell problem solution ($\chi _k$) in each direction for the full domain. **A** and **B** highlight the result for the small-scale corrector ($\chi _k$) along the *x*-direction. **C** and **D** highlight the result for the small-scale corrector along the *y*-direction. **E** and **F** highlight the result for the small-scale corrector along the *z*-direction

**Fig. 6:**
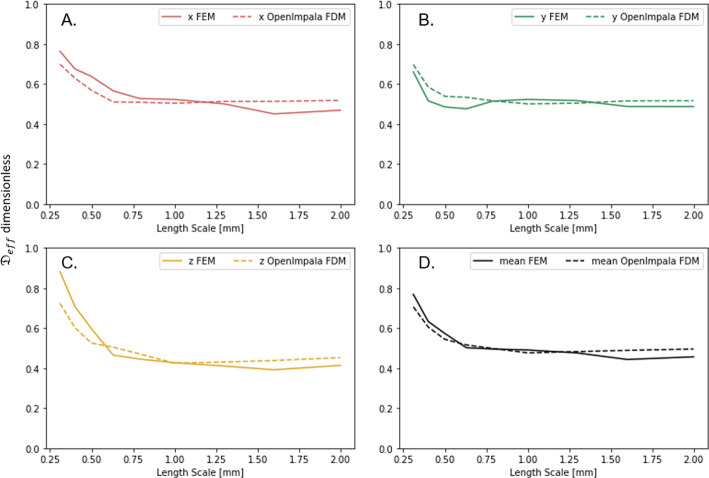
Comparison of the effective diffusivity obtained for different regions of interest via COMSOL FEM and OpenImpala FDM method. **A** compares the diffusivity component along the *x*-direction, **B** compares the diffusivity along the *y*-direction, **C** compares the diffusivity along the *z*-direction, and **D** compares the mean value of the diffusivity tensor for the two methods

Figure 5 illustrates the cell problem solutions ($\chi _k$) for the full image-based domain in each direction *k*. Results show both the variation of $\chi _k$ between the directions (*k*) and spatially within each solution. Due to the differences in solution between directions we expect the effective diffusion parameters (calculated by Eq. 15 using $\chi _k$) in each direction to vary, i.e. an-isotropic diffusion. The spatial variation within solutions highlights the importance of using a large enough EV so that when calculating ${\mathfrak {D}}_{eff}$ (Eq. 15, which can be thought of as performing an average on some linear function of $\chi _k$), the mean is representative of the material. The cell problem was solved for all sub EVs in order to obtain the effective diffusivity tensor at their respective scales (Fig. 6). The diagonals were solved using the OpenImpala FDM solver and the FEM solution. Diffusivity tensors were compared for the *x* component (Fig. 6A), *y* component (Fig. 6B), *z* component (Fig. 6C) and the overall tensor mean (Fig. 6D). The general trend is similar between the FDM and FEM results, with a rapid decay in the diffusivity values for all directions with increasing EV length scale. For the *x* component, *z* component, and overall mean diffusivity tensor, the FEM appears slightly larger than the FDM for the smallest EV. However, all of the diffusivity values appear slightly smaller for the FEM results at the larger EV length scale than the FDM. Both methods appear to converge near ${\mathfrak {D}}_{eff} \approx 0.4-0.6$.

**Fig. 7:**
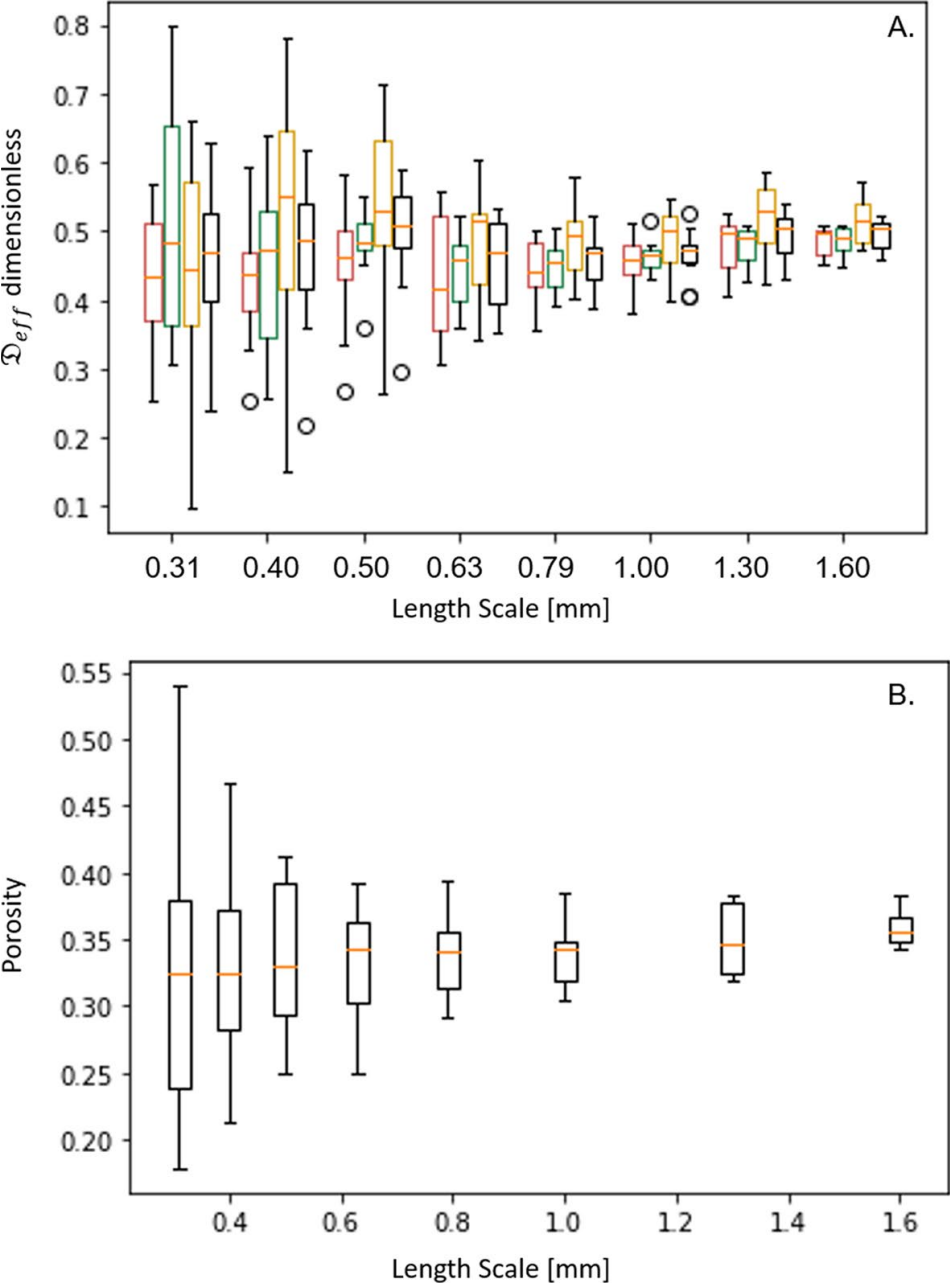
Comparison of **A** the effective diffusivity and **B** the porosity obtained for different subdomain sizes randomly positioned in the full domain. A subdomain/region of interest of a fixed size was placed in 10 random locations within the full domain

We used OpenImpala to test the variability of effective diffusivity and porosity values obtained for a given subdomain size by generating different regions of interest at random positions in the small-scale domain, as seen in Fig. 7. The results demonstrate that the ensemble mean of the randomly positioned regions of interest results in consistent values for the effective diffusivity parameter. However, the variability greatly diminishes with the increasing size of the region of interest, which is a key result. For a specific material, property or geometry, similar variability of effective parameter plots can be produced to determine the appropriate size of the representative elementary volume (REV). This functionality is expected to be used during imaging beamtimes to inform experimentalists of the statistical relevance of domain sizes.

Similar trends can be seen for the porosity (Fig. 7B). It is worth noting that the magnitude of the porosity is not sufficient for scaling the effective diffusivity, which is further discussed in Tjaden et al. (2016). Furthermore, geometric information of the pore space plays a role in illustrating differences in the effective diffusivity that would not be captured by only tracking the porosity.

### Comparing Image-Based Simulations to Uniform Domain Simulations that Consider Effective Parameters

**Fig. 8:**
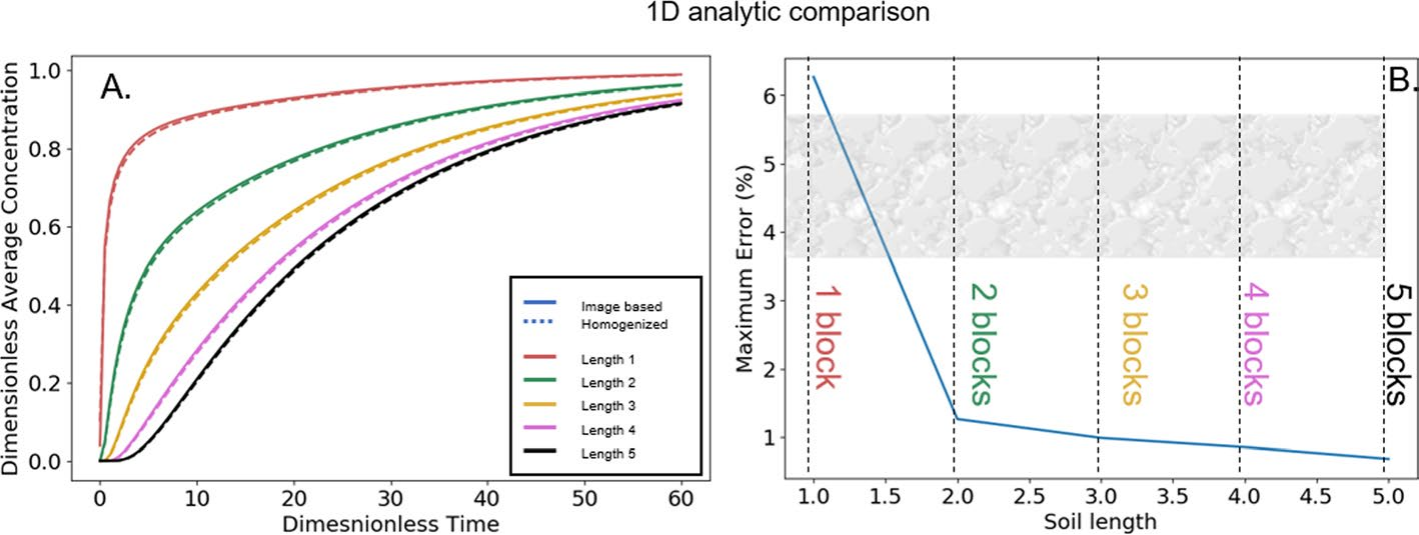
Comparison of results form an image-based model and a uniform 1D domain considering the homgenised effective diffusivity. **A** Time-dependent response highlights the average concentrations at the end of the respective blocks considering the full geometry and the uniform geometry using the homogenised effective diffusivity. **B** highlights the maximum error amongst the different block simulations from A, highlighting that the effective diffusivity falls below 1% after 3 blocks

The homogenisation procedure and the utility of the effective diffusivity were validated by comparing a 1D homogeneous-domain analytic solution using the effective diffusivity tensor to an image-based domain model using COMSOL (Fig. 8). The transient comparison between the averaged concentration at the end of each block for both methods highlights that the homogenised simulation slightly lags behind the image-based simulation for the single block and becomes similar in comparison for the increasing block lengths (Fig. 8A). Considering the maximum percentage difference at the end of each block over the duration of the simulations, we see that the percentage difference between the two simulations begins at around 6% for the single block (Fig. 8B). As we consider a larger domain size (2 blocks), the difference between the simulations drops to about 1.5 % (Fig. 8B). For larger domain sizes, the difference between the full image-based model and the homogenised uniform model drops below 1 % error (well below any experimental measurement error that you would find on a field site, Keller et al. (2017)), illustrating the utility of considering the homogenised model for domains exceeding the 3 blocks in length (Fig. 8B).

### Comparing Simulation Times Between OpenImpala and COMSOL

The cell problem was solved using OpenImpala and COMSOL on the IRIDIS5 high-performance computing facility in Southampton using a single node and 40 threads. The resulting COMSOL model required 102 min to solve the cell problem. By contrast, OpenImpala solved the cell problem in 15 s. Similar tests were conducted using 20 and 10 threads, with similar results. Further scaling performance for OpenImpala can be seen in Le Houx and Kramer (2021), detailing performance for larger domain sizes.

**Table 1:** Comparison of simulation times between OpenImpala and COMSOL to solve the cell problem for the largest domain size (8 × 10^6^ voxels)

Method	Threads / min				
	1 Thread	5 Threads	10 Threads	20 Threads	40 Threads
OpenImpala	3.07	0.70	0.39	0.28	0.19
COMSOL	150	82	73	80	100

**Fig. 9:**
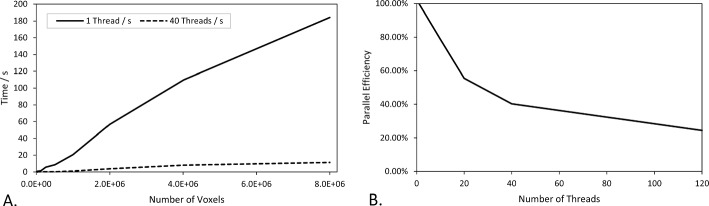
**A** Comparison of the time taken for OpenImpala to solve the cell problem across the eight domain sizes for single threaded computations, and 40 threads. **B** Parallel efficiency of OpenImpala for the 8 $\times$ 10^6^ voxel domain

## Discussion

In this study, we have updated the capabilities of OpenImpala (Le Houx and Kramer 2021) to account for more complex boundary conditions which can be used to estimate effective transport properties in porous media. Specifically, we have included flux modifications to the boundaries internal to the model domain and augmented the outer boundary conditions to consider periodic conditions for consistency with the homogenisation theory (i.e. Eq. 13). OpenImpala can now generate computationally fast estimates for effective diffusivity based on the geometry imaged at the synchrotron beamline using rigorous integration with asymptotic theoretical considerations (Hornung 1996).

To ensure the accuracy of the effective diffusivity estimates generated by OpenImpala, we compared OpenImpala’s results with a standard commercial FEM modelling package (COMSOL) for different EV domain sizes (Fig. 1B). The resulting effective diffusivity tensors (Fig. 6) highlighted similar trends in the values of tensor elements in various x,y, and z directions and the directional mean diffusivity (Fig. 6). All of the simulation results show a decay down to dimensionless values between 0.4$-$0.6. Considering that most nitrogen fertilisers (e.g. NO$^{-}_3$, NH$^{+}_4$) have diffusivity values in free water on the order of $1.8\times 10^{-9}$ m$^2$ s$^{-1}$ (Ruiz et al. (2020)), these nutrients would have effective diffusivity values on the order of $0.7-1.0\times 10^{-9}$ m$^2$ s$^{-1}$ due to geometric impedance in soil. The similarities in the results give us confidence that the estimates provided by OpenImpala are consistent with industry-standard packages like COMSOL. However, we subsequently ran simulations on larger domains to ensure that the effective diffusivity tensor predictions were accurate.

Simulations comparing larger domains with heterogeneous structure to the homogenised uniform 1D domains were carried out in COMSOL, as OpenImpala cannot currently carry out time-dependent simulations (Fig. 4). Using the previously determined effective diffusivity tensor, we demonstrated that differences between the structured and homogenised domains were within 7% error for a single domain size but rapidly dropped to about 1.5% when considering a domain size of two imaged units. After three blocks, the error dropped below 1%. In the context of soil science, even the most robust field-scale measurement systems are seldom capable of producing results at such low uncertainty values (Keller et al. 2017). Thus, for agricultural field practices, consideration of homogenised effective diffusivity is likely sufficient for practical use.

While this work has highlighted the utility of OpenImpala for general porous media transport studies, there are still certain restrictions that need to be overcome in the future. OpenImpala can only solve time independent, i.e. stationary, problems. While this was sufficient for estimating the effective diffusivity tensor, OpenImpala was limited in its ability to run verification independently. For a diffusion equation (no convection), an explicit time solver (i.e. Euler time stepping) might be sufficient. However, given the presence of advection, the solver will likely have to consider implicit time stepping approaches, such as Crank-Nicolson Crank and Nicolson (1947).

Another major use of multi-scale asymptotic homogenisation in porous media is quantifying hydraulic conductivity or permeability derived from Stokes flow (i.e. Darcy’s law (Daly and Roose 2014)). As Stokes flow is a steady-state equation, the OpenImpala solver can generate estimates of effective bulk-scale conductivity values. However, several equations would have to be introduced to account for the flow vector field and the pressure in a domain.

The effective diffusivity extracted in this work is a function of the geometric and topological information (Fig. 6), where for smaller sub-regions, we have higher porosity and thus higher effective diffusivity. The subdomains also have heterogeneity, which result in, albeit minor, anisotropy in our effective diffusivity tensor. Regarding multi-phase pore space domain, we can simplify diffusivity of inert solutes in fixed partially saturated regimes as single-phase transport by considering only the liquid phase. These model details can be found in Ruiz et al. (2020). While this work focused on validating the utility of rapidly extracting the influence of geometric impedance from an CT image, we understand that representative volumes may be different considering interactions between solutes and solid surfaces. Particular solute-surface reaction rates would play a role in how they influence representative volumes. Theoretically, many of these surface interactions would likely manifest as effective domain sources and sinks (see SI section 3 for details). However, if a solute is rapidly adsorbed and dissolved from surfaces at rates much quicker than the rates of transport, this could be simplified to another impedance term (formally considered as buffer power (Barber 1995), see SI section 4 for details). We endeavour to include these details in future versions of the model.

While the results from this study give us confidence in the estimated effective parameters generated by OpenImpala, we note that there were slight differences with some of the absolute values of the parameters (Fig. 6), with percent difference below 10%. Despite using the same imaged data set, the FEM simulations required a mesh instead of voxel images. As such, we generated a mesh from the segmented image stacks. This will produce a discrepancy between the two simulations, as the surfaces can be smoothed during segmentation and may no longer retain the full imaged information. However, FEM provides a more robust and reliable estimate for fluxes, given the weak-form treatment of the equations (Reddy 2019). Thus boundary conditions are likely better handled compared to the FDM employed by OpenImpala. These discrepancies are likely more relevant when considering the full heterogeneity of an image-based model. However, for estimating effective diffusivity values for the use of large-scale analysis, these differences are negligible.

A critical advantage that OpenImpala has over COMSOL for calculating effective transport properties, particularly, on site is the simulation speed, provided there is access to a parallel computing facility. Table 1 highlights the rapid solution time of OpenImpala, which was often two orders of magnitude faster than COMSOL. Furthermore, OpenImpala can be sped up further by increasing the thread count of parallelisation, whereas COMSOL appears to plateau after 10 threads. The speed of calculations is particularly important given that OpenImpala has to operate close to real time in the DIAD tomography processing pipeline. Figure 9 B. shows the parallel efficiency (Hill and Marty 2008) of OpenImpala as a function of thread count, for the largest domain size. Additionally, unlike finite element solvers such as COMSOL or finite volume solvers, OpenImpala can generate image-based models directly from segmented images, relinquishing the need for computationally expensive and technical meshing procedures. Lastly, OpenImpala is now tailored for effective parameter estimation, which facilitates its utility for non-modelling users. In conclusion, the rapid rate of computation, reliability of the results, ease of use, and seamless use of image data demonstrates OpenImpala’s capability for efficient on site effective parameter estimation, and thus its use as part of a semi-automated tomography processing pipeline.

## Supplementary Information

Below is the link to the electronic supplementary material.

Supplementary file1 (PDF 1096 KB)

Supplementary file2 (ZIP 1081 KB)

## Data Availability

The data is openly available at https://doi.org/10.5258/SOTON/D2453

## Code Availability

The software is publicly available at https://github.com/kramergroup/openImpala
