# Supplementary material for: Statistical Effective Diffusivity Estimation in Porous Media Using an Integrated On-site Imaging Workflow for Synchrotron Users
Source: Transp Porous Media. 2023 Jul 26;150(1):71–88. doi: 10.1007/s11242-023-01993-7 (PMC10468943; doi:10.1007/s11242-023-01993-7)
Supplement: Supplementary file 1 — Supplementary file1 (PDF 1096 KB) [file 11242_2023_1993_MOESM1_ESM.pdf]

Statistical Effective Diffusivity Estimation in Porous Media using an Integrated On-Site Imaging Workflow for Synchrotron Users - Supplementary Information

James Le Houx[1*†], Siul Ruiz[1†], Daniel McKay Fletcher[1,1†], Sharif Ahmed[1] and Tiina Roose[1]

*Corresponding author(s). E-mail(s): james.le-houx@diamond.ac.uk;
Contributing authors: s.a.ruiz@soton.ac.uk; dmmf1d19@soton.ac.uk; sharif.ahmed@diamond.ac.uk; t.roose@soton.ac.uk;
†These authors contributed equally to this work.

1 Effective Parameter Estimation

1.1 Mathematical Homogenisation Method - full derivation

Formal effective parameter estimation for different physical phenomena in porous media has been well established [1]. This study focuses on obtaining effective estimates for diffusivity initially considering an image based domain. A key assumption that will be followed in this work is that there exist a separation of scales associated with the small and large scale. The small scale presents all of the imaged detail in the pore space and is assumed to be periodic in the different directions. The large scale can take the estimated effective (or homogenised) diffusivity and generate estimates that are sufficient for describing the large transport scale behaviour without explicitly considering all of the pore scale detail.

Considering the diffusion equation:

$$\frac{\partial \tilde{c}}{\partial \tilde{t}} = \tilde{\nabla} \cdot (\tilde{D} \tilde{\nabla} \tilde{c}),\ \tilde{\mathbf{x}} \in \Omega, \tag{1}$$

where $\tilde{c}$ [mol m^{-3}] is a concentration of a transporting solute, $\tilde{t}$ [s] is time, $\tilde{D}$ [m^2 s^{-1}] is the diffusivity, $\tilde{\mathbf{x}}$ is the spatial coordinate associated with regions in the domain volume Ω (Figure 1 A.), and $\tilde{\nabla}$ is the spatial differentiation operator. Assuming the dimensional scaling of the different terms is

$$\begin{array}{ll} \tilde{\nabla}(\cdot) = \frac{1}{L_X}\nabla(\cdot), & \tilde{t} = \tau t \\ \tilde{c} = \bar{c}c, & \tilde{D} = \frac{L_x^2}{\tau} D \end{array}, \tag{2}$$

where L_X [m] is the large length scale, τ [s] is the time scale, and $\bar{c}$ is the average initial concentration of solute. The non-dimensional equation becomes:

$$\frac{\partial c}{\partial t} = \nabla \cdot (D \nabla c),\ \mathbf{x} \in \Omega. \tag{3}$$

We assume that the domain $\Omega = \bigcup_i^n \Omega_i$, where each Ω_i is a periodically repeating sub-domain (or cell) (Figure 1 A-B.). As such, the boundary condition for periodicity is given as:

$$c - \ periodic,\ \mathbf{x} \in \partial\Omega_i, \tag{4}$$

where $\partial\Omega_i$ is the outer boundary of a cell (Figure 1 B.). We assume that solutes cannot move across internal boundaries between pore-space and solids:

$$\hat{\mathbf{n}} \cdot (D \nabla c) = 0,\ \mathbf{x} \in \Gamma_i, \tag{5}$$

where Γ_i represents the internal boundaries between solids and pore-space (Figure 1 B.).

1.1.1 Multi-scale expansion

The dimensional differential operator can be expanded into a large scale estimate and a small scale corrector:

$$\tilde{\nabla}(\cdot) = \tilde{\nabla}_X(\cdot) + \tilde{\nabla}_\xi(\cdot), \tag{6}$$

where $\mathbf{X}$ and $\boldsymbol{\xi}$ are the large and small scale spatial variable respectively. The dimensional scaling of the differential operators is given by:

$$\tilde{\nabla}_X(\cdot) = \frac{1}{L_X}\nabla_X(\cdot),\ \tilde{\nabla}_\xi(\cdot) = \frac{1}{l_\xi}\nabla_\xi(\cdot)\,, \tag{7}$$

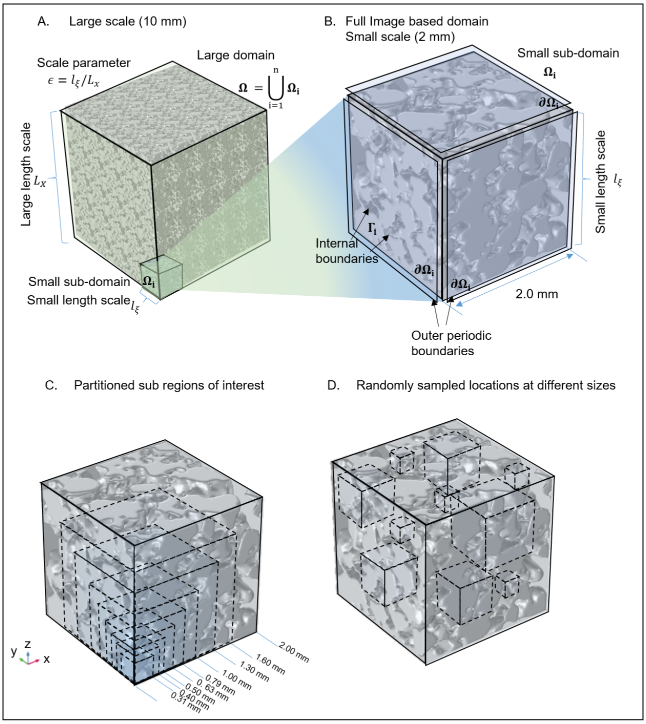

Fig. 1 Generating effective parameter estimate based on XCT images. A - Representation of a large scale pore space Ω comprised of periodic subdomains Ω_i. The length scale of the large domain is represented by L_X while the length scale of the subdomain is represented by l_ξ. B - The full image based geometry representing the pore space within a porous media (soil pore space in the given example). C - Determining the representative elementary volume sufficient for obtaining reasonable effective parameter (i.e. diffusivity) estimates. D - Generating a region of interest for a given size at a random location in the modelled domain.

Considering the relationship between the large scale and small scale as $L_X >> l_\xi$, we say that the ratio between the scales is given by $\frac{l_\xi}{L_x} = \epsilon$ where ϵ is a small number. Our dimensionless differential operator can be expressed as:

$$\nabla(\cdot) = \nabla_X(\cdot) + \epsilon^{-1}\nabla_\xi(\cdot). \tag{8}$$

We then assume that the concentration can be expanded as:

$$c = \epsilon^0 c_{(0)} + \epsilon^1 c_{(1)} + \epsilon^2 c_{(2)} + \mathcal{O}(\epsilon^3) \tag{9}$$

Substituting in eqs. 8 and 9 into eq. 3, we obtain the following equation on the cell:

$$\begin{aligned}\epsilon^0 \frac{\partial c_{(0)}}{\partial t} &= \epsilon^{-2}\nabla_\xi \cdot (D\nabla_\xi c_{(0)}) + \epsilon^{-1}(\nabla_\xi \cdot (D(\nabla_\xi c_{(1)} + \nabla_X c_{(0)})) \\ &+\nabla_X \cdot (D\nabla_\xi c_{(0)})) + \epsilon^0(\nabla_\xi \cdot (D(\nabla_\xi c_{(2)} + \nabla_X c_{(1)}))+ \\ &\nabla_X \cdot (D(\nabla_\xi c_{(1)} + \nabla_X c_{(0)})) + \mathcal{O}(\epsilon^1), \qquad \boldsymbol{\xi} \in \Omega_i,\end{aligned} \tag{10}$$

with the associated no flux conditions:

$$\begin{aligned}&\epsilon^{-2}\hat{\mathbf{n}}\cdot(D\nabla_\xi c_{(0)})+\epsilon^{-1}(\hat{\mathbf{n}}\cdot(D(\nabla_\xi c_{(1)}+\nabla_X c_{(0)})))\\&+\epsilon^0(\hat{\mathbf{n}}\cdot(D(\nabla_\xi c_{(2)}+\nabla_X c_{(1)})))+\mathcal{O}(\epsilon^1)=0, \qquad \boldsymbol{\xi}\in\Gamma_i,\end{aligned} \tag{11}$$

and

$$c_{(0)}, c_{(1)}, c_{(2)}, \ldots - \ \textit{periodic}, \ \boldsymbol{\xi}\in\partial\Omega_i. \tag{12}$$

We note that given the periodicity of the cell, it suffices to consider $D = D(\boldsymbol{\xi})$.

1.1.2 Order $\mathcal{O}(\epsilon^{-2})$

Considering only the terms in ϵ^{-2}, we have the following system of equations:

$$\begin{cases}\nabla_\xi\cdot(D\nabla_\xi c_{(0)})=0, & \boldsymbol{\xi}\in\Omega_i\\ \hat{\mathbf{n}}\cdot(D\nabla_\xi c_{(0)})=0, & \boldsymbol{\xi}\in\Gamma_i\\ c_{(0)}-\ \textit{periodic}, & \boldsymbol{\xi}\in\partial\Omega_i\end{cases}, \tag{13}$$

We consider the domain integral of $c_{(0)}(\nabla_\xi\cdot(D\nabla_\xi c_{(0)}))$ and use Green's identity to expand the integral and obtain:

$$\int_{\Omega_i} c_{(0)}(\nabla_\xi\cdot(D\nabla_\xi c_{(0)}))d\boldsymbol{\xi} = \oint_{\partial\Omega_i\cup\Gamma_i} c_{(0)}(\hat{\mathbf{n}}\cdot(D\nabla_\xi c_{(0)}))d\boldsymbol{\xi} - \int_{\Omega_i}(D\|\nabla_\xi c_{(0)}\|^2)d\boldsymbol{\xi}. \tag{14}$$

Due to the domain equations and boundary conditions (eq 13), only the last term remains. Thus, eq. 14 becomes:

$$\int_{\Omega_i}(D\|\nabla_\xi c_{(0)}\|^2)d\boldsymbol{\xi}=0. \tag{15}$$

This can only be true if either $D = 0$ or $\|\nabla_\xi c_{(0)}\|^2 = 0$. Assuming the non-trivial case (i.e. $D \neq 0$), we can deduce from this that $c_{(0)}(t,\boldsymbol{\xi},\mathbf{X}) = c_{(0)}(t,\mathbf{X})$, thus not varying on the small scale.

1.1.3 Order $\mathcal{O}(\epsilon^{-1})$

Considering the terms in ϵ^{-1}, we have the following system of equations:

$$\begin{cases}\nabla_\xi\cdot(D(\nabla_\xi c_{(1)}+\nabla_X c_{(0)}))+\nabla_X\cdot(D\nabla_\xi c_{(0)})=0, & \boldsymbol{\xi}\in\Omega_i\\ \hat{\mathbf{n}}\cdot(D(\nabla_\xi c_{(1)}+\nabla_X c_{(0)}))=0, & \boldsymbol{\xi}\in\Gamma_i\\ c_{(0)},c_{(1)}-\ \textit{periodic}, & \boldsymbol{\xi}\in\partial\Omega_i\end{cases}. \tag{16}$$

From eq 15, we can simplify the equation to:

$$\begin{cases}\nabla_\xi\cdot(D(\nabla_\xi c_{(1)}+\nabla_X c_{(0)}))=0, & \boldsymbol{\xi}\in\Omega_i\\ \hat{\mathbf{n}}\cdot(D(\nabla_\xi c_{(1)}+\nabla_X c_{(0)}))=0, & \boldsymbol{\xi}\in\Gamma_i\\ c_{(0)},c_{(1)}-\ \textit{periodic}, & \boldsymbol{\xi}\in\partial\Omega_i\end{cases}. \tag{17}$$

We represent the gradient term as:

$$\nabla_X c_{(0)} = \sum_i^n \hat{\mathbf{e}}_i \frac{\partial c_{(0)}}{\partial X_i}, \tag{18}$$

where $\hat{\mathbf{e}}_i$ are the unit vectors in the X_i directions, and we expand eq.17 as:

$$\begin{cases} \nabla_\xi \cdot (D\nabla_\xi c_{(1)}) = -\nabla_\xi \cdot (D(\sum_i^n \hat{\mathbf{e}}_i \frac{\partial c_{(0)}}{\partial X_i})), & \boldsymbol{\xi} \in \Omega_i \\ \hat{\mathbf{n}} \cdot (D(\nabla_\xi c_{(1)} + \sum_i^n \hat{\mathbf{e}}_i \frac{\partial c_{(0)}}{\partial X_i})) = 0, & \boldsymbol{\xi} \in \Gamma_i \\ c_{(0)}, c_{(1)} - \ periodic, & \boldsymbol{\xi} \in \partial\Omega_i \end{cases} . \tag{19}$$

We now consider the following system of equations who's solutions will act as small scale correctors to our homogenised system:

$$\begin{cases} \nabla_\xi \cdot (D\nabla_\xi \chi_i) = -\nabla_\xi \cdot (D\hat{\mathbf{e}}_i), & \boldsymbol{\xi} \in \Omega_i \\ \hat{\mathbf{n}} \cdot (D(\nabla_\xi \chi_i + \hat{\mathbf{e}}_i)) = 0, & \boldsymbol{\xi} \in \Gamma_i \\ \chi_i - \ periodic, & \boldsymbol{\xi} \in \partial\Omega_i \end{cases} , \tag{20}$$

where χ_i is the small scale corrector in the ith direction. If we multiply both sides of the eq. 20 by $\frac{\partial c_{(0)}}{\partial X_i}$, we obtain:

$$\begin{cases} \nabla_\xi \cdot (D\nabla_\xi \chi_i \frac{\partial c_{(0)}}{\partial X_i}) = -\nabla_\xi \cdot (D\hat{\mathbf{e}}_i \frac{\partial c_{(0)}}{\partial X_i}), & \boldsymbol{\xi} \in \Omega_i \\ \hat{\mathbf{n}} \cdot (D(\nabla_\xi \chi_i \frac{\partial c_{(0)}}{\partial X_i} + \hat{\mathbf{e}}_i \frac{\partial c_{(0)}}{\partial X_i})) = 0, & \boldsymbol{\xi} \in \Gamma_i \\ \chi_i \frac{\partial c_{(0)}}{\partial X_i} - \ periodic, & \boldsymbol{\xi} \in \partial\Omega_i \end{cases} . \tag{21}$$

Note that by eq. 15, $\frac{\partial c_{(0)}}{\partial X_i}$ is invariant to differentiation by the $\nabla_\xi(\cdot)$ operator. As such, if we take the summation of eq. 21 for the different i^{th} directions, we obtain:

$$\begin{cases} \nabla_\xi \cdot (D\nabla_\xi (\sum_i^n \chi_i \frac{\partial c_{(0)}}{\partial X_i})) = -\nabla_\xi \cdot (D(\sum_i^n \hat{\mathbf{e}}_i \frac{\partial c_{(0)}}{\partial X_i})), & \boldsymbol{\xi} \in \Omega_i \\ \hat{\mathbf{n}} \cdot (D(\nabla_\xi (\sum_i^n \chi_i \frac{\partial c_{(0)}}{\partial X_i}) + \sum_i^n \hat{\mathbf{e}}_i \frac{\partial c_{(0)}}{\partial X_i})) = 0, & \boldsymbol{\xi} \in \Gamma_i \\ \sum_i^n \chi_i \frac{\partial c_{(0)}}{\partial X_i} - \ periodic, & \boldsymbol{\xi} \in \partial\Omega_i \end{cases} . \tag{22}$$

By substituting eq. 22 into eq. 19, we can see the relationship between the two scales manifests as:

$$c_{(1)}(t, \boldsymbol{\xi}, \mathbf{X}) = \sum_{i=1}^n \chi_i(\boldsymbol{\xi}) \frac{\partial c_{(0)}(t, \mathbf{X})}{\partial X_i} + \bar{c}_{(1)}(t, \mathbf{X}), \tag{23}$$

where $\bar{c}_{(1)}$ vanishes under differentiation by the $\nabla_\xi(\cdot)$ operator.

1.1.4 Order $\mathcal{O}(\epsilon^0)$

We consider lastly the terms on the order $\mathcal{O}(\epsilon^0)$:

$$\begin{cases} \frac{\partial c_{(0)}}{\partial t} = \nabla_\xi \cdot (D(\nabla_\xi c_{(2)} + \nabla_X c_{(1)})) + \nabla_X \cdot (D(\nabla_\xi c_{(1)} + \nabla_X c_{(0)})), & \boldsymbol{\xi} \in \Omega_i \\ \hat{\mathbf{n}} \cdot (D(\nabla_\xi c_{(2)} + \nabla_X c_{(1)})) = 0, & \boldsymbol{\xi} \in \Gamma_i \\ c_{(0)}, c_{(1)}, c_{(2)} - \ periodic, & \boldsymbol{\xi} \in \partial\Omega_i \end{cases} . \tag{24}$$

We consider the domain equation inside of the integral. As such, we have:

$$\int_{\Omega_i} \frac{\partial c_{(0)}}{\partial t} d\boldsymbol{\xi} = \int_{\Omega_i} (\nabla_\xi \cdot (D(\nabla_\xi c_{(2)} + \nabla_X c_{(1)})) + \nabla_X \cdot (D(\nabla_\xi c_{(1)} + \nabla_X c_{(0)})))d\boldsymbol{\xi}. \tag{25}$$

By divergence theorem, we can see that the first term becomes:

$$\int_{\Omega_i} (\nabla_\xi \cdot (D(\nabla_\xi c_{(2)} + \nabla_X c_{(1)})))d\boldsymbol{\xi} = \oint_{\partial\Omega_i \cup \Gamma_i} (\hat{\mathbf{n}} \cdot (D(\nabla_\xi c_{(2)} + \nabla_X c_{(1)})))d\boldsymbol{\xi}, \tag{26}$$

and by the boundary conditions in eq. 24, we can see:

$$\oint_{\partial\Omega_i \cup \Gamma_i} (\hat{\mathbf{n}} \cdot (D(\nabla_\xi c_{(2)} + \nabla_X c_{(1)})))d\boldsymbol{\xi} = 0. \tag{27}$$

Thus, eq. 25 can be re-written as:

$$\int_{\Omega_i} \frac{\partial c_{(0)}}{\partial t} d\boldsymbol{\xi} = \int_{\Omega_i} (\nabla_X \cdot (D(\nabla_\xi c_{(1)} + \nabla_X c_{(0)})))d\boldsymbol{\xi}. \tag{28}$$

By substituting in eq. 23 into eq. 28, we get:

$$\int_{\Omega_i} \frac{\partial c_{(0)}}{\partial t} d\boldsymbol{\xi} = \int_{\Omega_i} (\nabla_X \cdot (D(\nabla_\xi \chi_k \otimes \hat{\mathbf{e}}_k + \mathbf{I})\nabla_X c_{(0)}))d\boldsymbol{\xi}, \tag{29}$$

where $\mathbf{I}$ is the n sized identity matrix and $\nabla_\xi \chi_k \otimes \hat{\mathbf{e}}_k = \sum_{i=1}^{n} \nabla_\xi \chi_i \hat{\mathbf{e}}_i^T$. Given that $c_{(0)}$ is does not vary on Ω_i and $\nabla_X(\cdot)$ is separate from $\boldsymbol{\xi}$, eq. 29 can be re-written as:

$$\|\Omega_i\| \frac{\partial c_{(0)}}{\partial t} = \nabla_X \cdot (\int_{\Omega_i} (D(\nabla_\xi \chi_k \otimes \hat{\mathbf{e}}_k + \mathbf{I}))d\boldsymbol{\xi} \nabla_X c_{(0)}), \tag{30}$$

were $\|\Omega_i\| = \int_{\Omega_i} d\boldsymbol{\xi}$. Thus, the large scale homogenised diffusion equations can be represented as:

$$\frac{\partial c_{(0)}}{\partial t} = \nabla_X \cdot (\mathfrak{D}_{eff} \nabla_X c_{(0)}), \quad \mathbf{X} \in \Omega, \tag{31}$$

where

$$\mathfrak{D}_{eff} = \frac{1}{\|\Omega_i\|} \int_{\Omega_i} (D(\nabla_\xi \chi_k \otimes \hat{\mathbf{e}}_k + \mathbf{I}))d\boldsymbol{\xi}, \tag{32}$$

is the effective diffusivity parameter.

2 1D analytic solution used for validation

To validate the results of our effective diffusivity parameter, we ran numerical simulations with the full heterogeneous geometry duplicated in a horizontal array and compared the results to a 1 D analytic solution of the same length scale uniform geometry considering the effective diffusivity. The 1D model considered the following equations:

$$\begin{cases} \frac{\partial c_{(0)}}{\partial t} = \mathfrak{D}_{eff} \frac{\partial^2 c_{(0)}}{\partial X^2}, & X \in \Omega \\ c_{(0)} = 0, & t = 0, X \in \Omega \\ c_{(0)} = c_\infty, & X = 0 \\ \mathfrak{D}_{eff} \frac{\partial c_{(0)}}{\partial X} = 0, & X = L \end{cases}. \tag{33}$$

We define a variable $\hat{c}_{(0)} = c_{(0)} - c_\infty$, where we can re-write our equation as:

$$\begin{cases} \frac{\partial \hat{c}_{(0)}}{\partial t} = \mathfrak{D}_{eff} \frac{\partial^2 \hat{c}_{(0)}}{\partial X^2}, & X \in \Omega \\ \hat{c}_{(0)} = -c_\infty, & t = 0, X \in \Omega \\ \hat{c}_{(0)} = 0, & X = 0 \\ \frac{\partial \hat{c}_{(0)}}{\partial X} = 0, & X = L \end{cases}. \tag{34}$$

We define $\hat{c}_{(0)}(t, X) = Y(X)T(t)$, which allows us to use separation of variables. We can re-write the domain equation of eq 34 as:

$$T'(t)Y(X) = \mathfrak{D}_{eff} T(t) Y''(X), \tag{35}$$

which can then be expressed as:

$$\frac{T'(t)}{\mathfrak{D}_{eff} T(t)} = \frac{Y''(X)}{Y(X)} = -\lambda, \tag{36}$$

where λ is a constant. This generates two ordinary differential equations, one in space:

$$\begin{cases} Y''(X) + \lambda Y(X) = 0 \\ Y(0) = 0 \\ Y'(L) = 0 \end{cases}, \tag{37}$$

and one in time:

$$T'(t) + \lambda \mathfrak{D}_{eff} T(t) = 0. \tag{38}$$

Considering the Boundary value problem in eq. 37, our general solution will take the form:

$$Y(X) = A\cos(\sqrt{\lambda}X) + B\sin(\sqrt{\lambda}X). \tag{39}$$

Given the first boundary condition in eq. 37, we know that $A = 0$. If we differentiate the solution in eq. 39 and use the second boundary condition in eq. 37, we get the expression:

$$Y'(L) = \sqrt{\lambda} B \sin(\sqrt{\lambda} L) = 0, \tag{40}$$

which implies

$$\lambda = \lambda_n = (\frac{\pi}{2L}(2n+1))^2. \tag{41}$$

Therefore, we have

$$Y_n(X) = \sin(\frac{\pi}{2L}(2n+1)X). \tag{42}$$

Returning to eq. 38, we know that the general solution will be of the form:

$$T_n(t) = c_n \exp(-\lambda_n \mathfrak{D}_{eff} t). \tag{43}$$

Our solution can be a super position of solutions, thus $\hat{c}_{(0)}(t, X) = \sum_{n=0}^{\infty} Y_n(X) T_n(t)$ expands explicitly to

$$\hat{c}_{(0)}(t, X) = \sum_{n=0}^{\infty} c_n \exp(-(\frac{\pi}{2L}(2n+1))^2 \mathfrak{D}_{eff} t) \sin(\frac{\pi}{2L}(2n+1)X). \tag{44}$$

Given the initial condition of eq. 34, we know:

$$\sum_{n=0}^{\infty} c_n \sin(\frac{\pi}{2L}(2n+1)X) = -c_\infty. \tag{45}$$

Using the the properties of orthogonality, we know that $\langle \sin(\sqrt{\lambda_m}X), \sin(\sqrt{\lambda_n}X)\rangle = 0$, and $\langle \sin(\sqrt{\lambda_n}X), \sin(\sqrt{\lambda_n}X)\rangle = L$. We use this to solve for the unknown coefficient:

$$c_n = -\frac{2}{L}\int_0^L c_\infty \sin(\sqrt{\lambda_n}X) dX. \tag{46}$$

Thus, $c_n = -\frac{4c_\infty}{\pi(2n+1)}$. Since our full equation is $c_{(0)} = c_\infty + \hat{c}_{(0)}$, we have our analytic homogenised estimate as:

$$c_{(0)}(t, X) = c_\infty(1 - \sum_{n=0}^{\infty} \frac{4}{\pi(2n+1)} e^{-\mathfrak{D}_{eff}(\frac{\pi}{2L}(2n+1))^2 t} \sin(\frac{\pi}{2L}(2n+1)X)). \tag{47}$$

3 Influence of surface reactions on homogenisation

While the scope of this study was to investigate the influence of physical geometric impedance on mass transport, we know that the homogenisation

method can also support estimates for binding and dissolution of solutes from surfaces. We revisit the dimensional form of our equations but also consider surface interactions:

$$\begin{cases} \frac{\partial \tilde{c}}{\partial \tilde{t}} = \tilde{\nabla} \cdot (\tilde{D}\tilde{\nabla}\tilde{c}), & \tilde{\mathbf{x}} \in \Omega_i \\ \frac{\partial \tilde{c}_s}{\partial \tilde{t}} = -k_d \tilde{c}_s + U\tilde{c}, & \tilde{\mathbf{x}} \in \Gamma_i \\ \hat{\mathbf{n}} \cdot (\tilde{D}\tilde{\nabla}\tilde{c}) = (k_d \tilde{c}_s - U\tilde{c}), & \tilde{\mathbf{x}} \in \Gamma_i \end{cases}, \tag{48}$$

where $\tilde{c}_s$ [mol m^{-2}] is the surface bound concentrations, k_d [s^{-1}] is the dissolution rate, U [m s^{-1}] is the binding rate. We consider all of the same scaling arguments from eqs. 2, 7, and 8 along with a scaling for bound concentrations $\tilde{c}_s = \bar{c} l_\xi c_s$. We can re-write eq. 48 as:

$$\begin{cases} \frac{\partial c}{\partial t} = \nabla \cdot (D\nabla c), & \mathbf{x} \in \Omega_i \\ \frac{\partial c_s}{\partial t} = -\tau(k_d c_s - \frac{U}{l_\xi} c), & \mathbf{x} \in \Gamma_i \\ \hat{\mathbf{n}} \cdot (D\nabla c) = \tau\epsilon(k_d c_s - \frac{U}{l_\xi} c), & \tilde{\mathbf{x}} \in \Gamma_i \end{cases}. \tag{49}$$

We follow the same expansions in eq. 9 (also for c_s), and we find that $\mathcal{O}(\epsilon^{-2})$ and $\mathcal{O}(\epsilon^{-1})$ eqs. work out to be identical to what we've had without the reaction terms. However, $\mathcal{O}(\epsilon^0)$ has changed:

$$\begin{cases} \frac{\partial c_{(0)}}{\partial t} = \nabla_\xi \cdot (D(\nabla_\xi c_{(2)} + \nabla_X c_{(1)})) + \nabla_X \cdot (D(\nabla_\xi c_{(1)} + \nabla_X c_{(0)})), & \boldsymbol{\xi} \in \Omega_i \\ \hat{\mathbf{n}} \cdot (D(\nabla_\xi c_{(2)} + \nabla_X c_{(1)})) = \tau\ (k_d c_{(0),s} - \frac{U}{l_\xi} c_{(0)}), & \boldsymbol{\xi} \in \Gamma_i \\ \frac{\partial c_{(0),s}}{\partial t} = -\tau\ (k_d c_{(0),s} - \frac{U}{l_\xi} c_{(0)}), & \boldsymbol{\xi} \in \Gamma_i \\ c_{(0)}, c_{(1)}, c_{(2)} -\ periodic, & \boldsymbol{\xi} \in \partial\Omega_i \\ c_{(0),s}, c_{(1),s}, c_{(2),s} -\ periodic, & \boldsymbol{\xi} \in \partial\Omega_i \end{cases}. \tag{50}$$

We consider the domain equation under integration as done before in eq. 25, and using divergence theorem as done in eq. 26, we instead see:

$$\oint_{\partial\Omega_i \cup \Gamma_i} (\hat{\mathbf{n}} \cdot (D(\nabla_\xi c_{(2)} + \nabla_X c_{(1)})))d\boldsymbol{\xi} = \tau \oint_{\Gamma_i} (k_d c_{(0),s} - \frac{U}{l_\xi} c_{(0)})d\boldsymbol{\xi}. \tag{51}$$

We can express the integral equation as:

$$\|\Omega_i\| \frac{\partial c_{(0)}}{\partial t} = \nabla_X \cdot (\int_{\Omega_i} (D(\nabla_\xi \chi_k \otimes \hat{\mathbf{e}}_k + \mathbf{I}))d\boldsymbol{\xi} \nabla_X c_{(0)}) + \tau \oint_{\Gamma_i} (k_d c_{(0),s} - \frac{U}{l_\xi} c_{(0)})d\boldsymbol{\xi}, \tag{52}$$

Given that $c_{(0)}$ doesn't vary on the ξ scale and assuming that $c_{(0),s}$ behaves the same, we can express the equation as:

$$\|\Omega_i\|\frac{\partial c_{(0)}}{\partial t} = \nabla_X \cdot \left(\int_{\Omega_i} (D(\nabla_\xi \chi_k \otimes \hat{\mathbf{e}}_k + \mathbf{I}))d\boldsymbol{\xi}\nabla_X c_{(0)}\right) + \tau\|\Gamma_i\|(k_d c_{(0),s} - \frac{U}{l_\xi}c_{(0)}), \tag{53}$$

where $\oint_{\Gamma_i} d\boldsymbol{\xi} = \|\Gamma_i\|$. Finally, we can come to our effective homogenised equation considering the surface reactions:

$$\frac{\partial c_{(0)}}{\partial t} = \nabla_X \cdot (\mathfrak{D}_{eff}\nabla_X c_{(0)}) + \mathfrak{R}_{eff}, \quad \mathbf{X} \in \Omega, \tag{54}$$

where

$$\mathfrak{R}_{eff} = \tau\frac{\|\Gamma_i\|}{\|\Omega_i\|}(k_d c_{(0),s} - \frac{U}{l_\xi}c_{(0)}), \tag{55}$$

is the effective reaction source/sink term that act on the domain.

4 Rapid reaction rates and buffer power

A common assumption used in soil science is that the reaction rates occur more rapidly than the transport timescale. We consider a small scale factor $\delta << 1$. If we assume $k_d = (\delta\tau)^{-1}\hat{k}_d$ and $U = (\delta\tau)^{-1}\hat{U}$. We re-write the eq. 55 to:

$$\mathfrak{R}_{eff} = \delta^{-1}\frac{\|\Gamma_i\|}{\|\Omega_i\|}(\hat{k}_d c_{(0),s} - \frac{\hat{U}}{l_\xi}c_{(0)}) = \delta^{-1}(\mathfrak{K}_d c_{(0),s} - \mathfrak{K}_a c_{(0)}), \tag{56}$$

which yields:

$$\begin{cases} \frac{\partial c_{(0)}}{\partial t} = \nabla_X \cdot (\mathfrak{D}_{eff}\nabla_X c_{(0)}) + \delta^{-1}(\mathfrak{K}_d c_{(0),s} - \mathfrak{K}_a c_{(0)}), & \mathbf{X} \in \Omega \\ \frac{\|\Gamma_i\|}{\|\Omega_i\|}\frac{\partial c_{(0),s}}{\partial t} = -\delta^{-1}(\mathfrak{K}_d c_{(0),s} - \mathfrak{K}_a c_{(0)}), & \mathbf{X} \in \Omega \end{cases}. \tag{57}$$

The bulk $\mathcal{O}(\delta^{-1})$ time scale, we can see:

$$c_{(0),s} = \frac{\mathfrak{K}_a}{\mathfrak{K}_d}c_{(0)}. \tag{58}$$

On the $\mathcal{O}(\delta^0)$ time scale, combining the two equations in eq. 57 yields:

$$\frac{\partial c_{(0)}}{\partial t} + \frac{\|\Gamma_i\|}{\|\Omega_i\|}\frac{\partial c_{(0),s}}{\partial t} = \nabla_X \cdot (\mathfrak{D}_{eff}\nabla_X c_{(0)}), \quad \mathbf{X} \in \Omega. \tag{59}$$

Substituting eq. 58 into eq. 59 yields:

$$(1 + \mathfrak{b})\frac{\partial c_{(0)}}{\partial t} = \nabla_X \cdot (\mathfrak{D}_{eff}\nabla_X c_{(0)}), \quad \mathbf{X} \in \Omega, \tag{60}$$

where $\mathfrak{b} = \frac{\|\Gamma_i\|}{\|\Omega_i\|} \frac{\mathfrak{K}_a}{\mathfrak{K}_d}$ is considered the buffer power [2]. Under these conditions, the time scales can influence the representative elementary volumes (REVs). However, quantification of the influence of reaction rates on REVs extended beyond the scope of the current study.

References

[1] Hornung, U.: Homogenization and Porous Media. Springer, 6 (1996)

[2] Barber, S.A.: Soil Nutrient Bioavailability: a Mechanistic Approach. John Wiley & Sons, ISBN:0471587478 (1995)
