# Supplementary figures and images for: Statistical Effective Diffusivity Estimation in Porous Media Using an Integrated On-site Imaging Workflow for Synchrotron Users
Source: Transp Porous Media. 2023 Jul 26;150(1):71–88. doi: 10.1007/s11242-023-01993-7 (PMC10468943; doi:10.1007/s11242-023-01993-7)

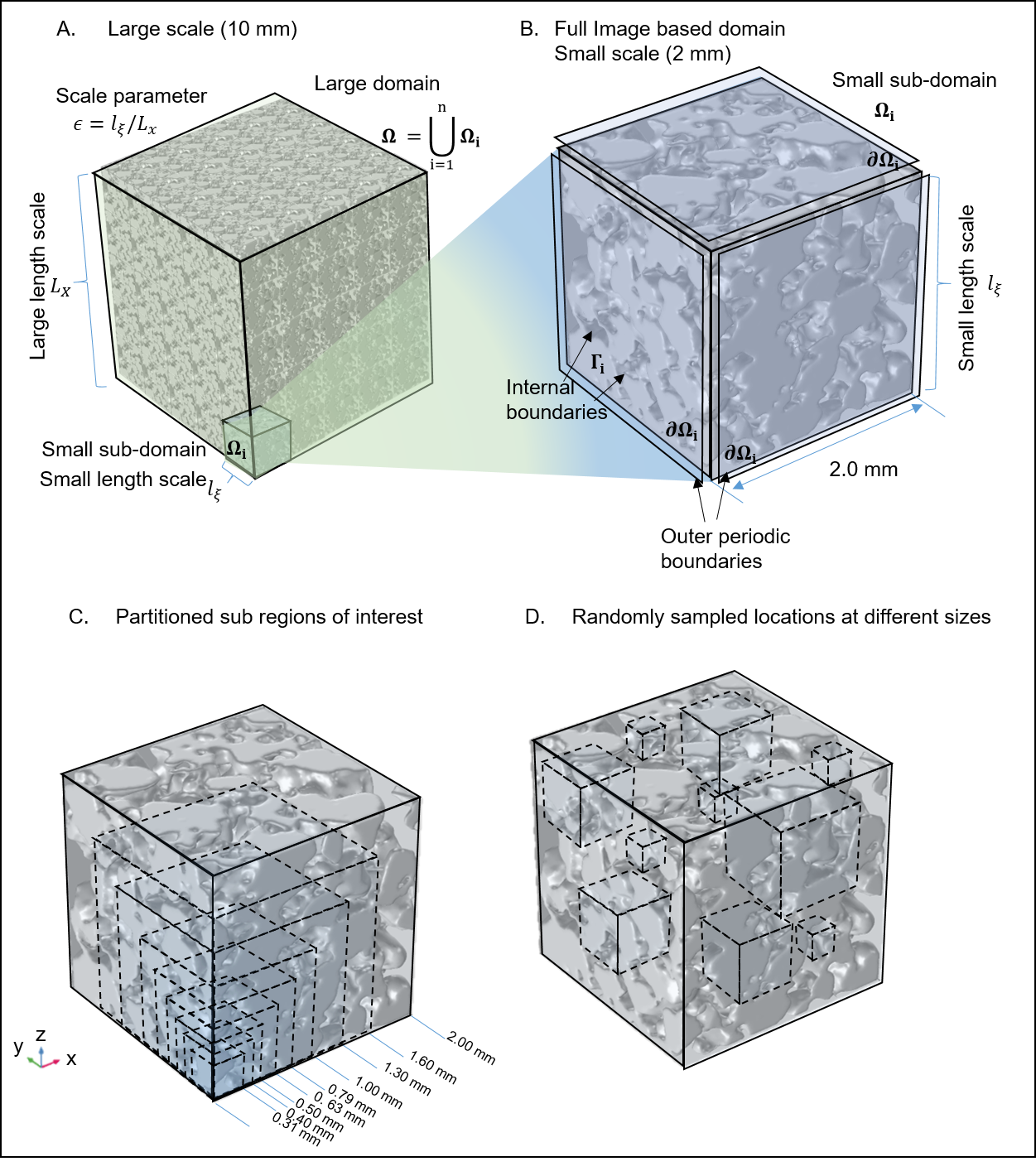

Supplement: Supplementary file 2 — Supplementary file2 (ZIP 1081 KB) [file 11242_2023_1993_MOESM2_ESM.zip › ROI_v4.png]
